# The influence of the ’good’ patient ideal on engagement in HIV care

**DOI:** 10.1371/journal.pone.0214636

**Published:** 2019-03-28

**Authors:** Kimberly A. Koester, Mallory O. Johnson, Troy Wood, Rob Fredericksen, Torsten B. Neilands, John Sauceda, Heidi M. Crane, Michael J. Mugavero, Katerina A. Christopoulos

**Affiliations:** 1 Department of Medicine, Center for AIDS Prevention Studies, University of California, San Francisco, California, United States of America; 2 Department of Medicine, University of Washington, Seattle, Washington, United States of America; 3 Department of Medicine, University of Alabama, Birmingham, Alabama, United States of America; Agencia de Salut Publica de Barcelona, SPAIN

## Abstract

Research on patient engagement in health care shows that better health outcomes and lower healthcare costs are observed among highly engaged patients. Similar to other illnesses, high levels of patient engagement in HIV care are considered essential to maintaining optimal health, and patients who are on treatment and retained in HIV care are known to have better health outcomes. In this article, we draw on focus group discussion data with patients living with HIV in order to explain tacit expectations associated with engagement in care. The main objective of our research was to elicit an explanatory model of engagement in HIV care from the patients’ perspective. We conducted focus group discussions with a sample of two distinct types of patients: those who regularly attended medical appointments and those who did not. In total, we conducted six focus group discussions (*n* = 43) across in three cities in the US; these included two focus group discussions with a well-engaged and less-well-engaged group in each location. Both types of patients assigned a moral dimension to engagement in care, in that well-engaged patients were considered to be ‘good’ patients. Aspiring to become a ‘good’ patient provided a meaningful goal for some and deepened vulnerabilities among patients that struggled to achieve this status. More vulnerable patients may feel less secure in health care interactions and these feelings may be amplified if patients have an unreasonable impression of what constitutes a ‘good’ patient; thereby leading to disengagement in care. Our findings can inform the development of patient-centered, tailored messages to better serve patients struggling to stay engaged in HIV care.

## Introduction

Patient engagement in care is a growing topic of interest commensurate with an increasing emphasis on patient-centered care [1–5]. Research on patient engagement shows that better health outcomes and lower healthcare costs are observed among highly engaged patient populations [6–10]. A high degree of patient engagement is therefore desirable, and healthcare experts are seeking ways to increase patient engagement across a range of medical settings and conditions [1,11].

In spite of the importance of patient engagement, there has been no unifying definition of the meaning ofpatient engagement. Generally speaking, patient engagement refers to the degree to which patients proactively invest in their health care. Recently Higgins and colleagues published the results of an extensive concept analysis of patient engagement [2,12] and provided a comprehensive definition, perhaps the most unifying to date: “…the desire and capability to actively choose to participate in care in a way uniquely appropriate to the individual in cooperation with a healthcare provider or institution for the purposes of maximizing outcomes or experiences of care”[2]. Several key attributes associated with patient engagement were identified and included personalization of the approach to care, access to necessary resources, commitment to pursuing quality care and therapeutic alliance.

Similarly, engagment in HIV care has become a critical issue to address as the challenges associated with HIV have shifted from acute to chronic disease management. As with other chronic diseases, high levels of patient engagement in HIV care are considered essential to the optimization of HIV treatment [13–15]. Patients who are on treatment and retained in care have better health outcomes [16]. Ideally, people living with HIV initiate treatment immediately following HIV diagnosis and remain retained in HIV care over their lifespan, an outcome that has proved tdifficult to achieve [17–19].

In addition to the key attributes as identified by Higgins and colleagues, much work has been done to identify the barriers and facilitators to engagement in HIV care. For example, patient-provider interactions, communication, and the presence or absence of trust are known to significantly impact engagement in care (see for example, [20–25]). A recent meta-analysis of qualitative studies identified the intrapersonal factors that are essential to engagement in care. These factors include, the importance of a person’s psychological state when diagnosed and soon after, as well as burn out or fatigue associated with managing healthcare for a chronic condition over time [26]. Health care delivery systems have been responsive to the need for greater interventions to help connect people newly diagnosed with HIV and those disengaged from care through the provision of logistical and emotional support such as appointment reminders or access to linkage to care specialists and/or patient navigators. Yet further research is needed to identify interventions to assist patients to successfully adapt to, integrate and accept one’s HIV diagnosis.

To date, little research exists describing how *patients* define engagement in HIV care or what being “engaged” means for them. Our team has through qualitative work with PLWH described a pattern of shifting attitudes towards one’s care, including having health concerns that may fade over time, taking ownership over one’s health and moving towards shared decision-making with providers, and recognizing appointments may decrease over time [27]. We also describe acceptance of HIV status as a key shift having occurred among a sample of patients who were retained in care [27]. Qualitative research has also demonstrated that patients who are deemed “out-of-care” by clinical definitions may not share that perspective [28]. These findings led to recommendations of *patient-centered* definitions of care engagement—that is to allow the patient to define the parameters of care engagement rather than to use metrics defined by appointment or viral load status. Building on this work, we examined perspectives on care engagement among patients who regularly attend clinic (retained in care) and those who do not (less-well-retained in care) as part of a larger research project to develop a quantitative index measure of engagement in HIV care [29]. Our research differs from earlier literature in that we asked focus group participants to generate definitions of the engaged and not well engaged patient which allowed us to explore the underlying meanings of these concepts. In this analysis, we describe how these tacit meanings shape patient engagement in care.

## Methods

The University of California San Francisco Campus (UCSF) Human Research Protection Program (HRPP) served as the IRB of record and approved all study procedures. Additionally, all study procedures were approved by the Institutional Review Board at the University of Alabama Birmingham and the University of Washington. Written or verbal informed consent was obtained from all participants prior to participation in the study. A full description of the consent process is included in the section below.

We conducted six focus group discussions (FGD) with patients currently receiving care at an HIV clinic affiliated with an academic medical center in Birmingham, Alabama, San Francisco, California, and Seattle, Washington.

### Sample, Eligibility and Recruitment

Patients were eligible for FGDs if they were 1) diagnosed with HIV and currently a patient at one of three Center for AIDS Research (CFAR) Network of Integrated Clinical Systems (CNICS) HIV clinic sites (in AL, CA, WA); 2) in HIV care for at least 12 months; and 3) ≥ 18 years old (≥19+ for AL participants). The total sample was purposefully sampled so that half of the FGD participants were considered to be optimally retained in HIV care, defined as: 1) no missed medical appointments in the past 12 months; 2) met the Health Service and Resource Administration (HRSA) definition of retention in care of two visits at least 90 days apart in the past 12 months; and 3) had an undetectable HIV viral load. The other half were considered to be sub-optimally retained in HIV care, which was defined as at least two or more missed HIV medical appointments in the past 12 months, or failure to meet the HRSA measure of retention. We refer to these two halves of the sample as “retained” and “less-well-retained.”

Participants in WA and AL were recruited by clinic staff at each of the clinical sites as they were completing a standardized health assessment. In California, HIV providers were also asked to identify potentially eligible patients via email and at staff meetings and were encouraged to refer retained and less-well-retained patients. Once a pool of potential participants was identified, study staff from each site reviewed patients’ medical records to ensure they met the eligibility criteria defined above.

### Procedures

We held 2 FGDs in each of the three locations (one focused on retained patients and one on less well-retained patients) in a private area in or near the clinic. We provided refreshments and $50 in cash to participant to offset the cost of travel to the clinic and to reimburse them for their time. Authors KK and RF are expert qualitative researchers and served as co-facilitators of the FGD. We invited a member of the local research team, in most cases a researcher with experience in participant-observation, to serve as a note taker (eg author TW in the San Francisco site). At the beginning of each group, we introduced ourselves, provided an overview of the study and goals of the focus group, described our respective roles and laid out the expections and structure of the group e.g., asked participants to avoid talking over one another, take breaks as needed. In an effort to build rapport and trust, the FGD team members each briefly discussed their history in conducting work with people living with HIV. We followed a semi-structured discussion guide that covered the following domains: health and health care experiences, definition of the engaged and not well engaged patient, engagement in care, and the role of the provider/clinic on engagement in care. In terms of group facilitation, at times, either RF or KK directly asked a member of the group to share their opinion on a topic in a round robin fashion, otherwise we encouraged free flowing responses and discussion among the group members. When eliciting narratives about the concept of engagement in care, we asked different variations on the same theme: what does engagement in care mean to you? How do you define an engaged or not well engaged patient? How engaged do you see yourselves in health care? Each participant provided informed consent prior to the start of the focus group and filled out a short demographic survey following the FGD. We held a debriefing session to identify key themes and to discuss any adjustments that would improve the facilitation of future FGDs. Typically KK or RF drafted a field note describing the content of the group, instances of non-verbal communication, and initial impressions of key ideas, which was then distributed to the wider team for review and discussion. Each FGD was digitally recorded using two recorders. We had these audio files professionally transcribed verbatim, including assigning names to each speaker.

### Analysis

Because our focus group discussion data were gathered as part of a larger study with a multidisciplinary set of investigators, we utilized the Framework Analysis approach to identify themes within the data set [30]. This analytic approach includes well-defined procedures including data familiarization, sorting, charting, and interpreting. We find this systematic analytic approach to be useful when working collaboratively on a study consisting of team members with different disciplinary backgrounds, as it helps to build confidence in the qualitative research findings.At any point, we could inform the larger team about the analytic step we were working on and could produce the outputs of our efforts along the different phases of analysis. During the familiarization phase, the primary analyst (KK) drafted a series of analytic memos summarizing the key points based on transcripts and field notes associated with each site. The site-specific memos compared and contrasted the perspectives of the retained and less well-retained participants producing a within-case analysis [31]. Following the review of site-specific memos, KK grouped the perspectives of all retained participants and compared these to the less well-retained participants to produce an across-case analysis. The transcripts, field notes, and analytic memos were entered into *Dedoose*, an online qualitative data management application [32]. We then sorted or applied a deductive coding frame consisting of the domains from the FGD guide to the transcripts. All excerpts associated with engagement in care narratives were extracted for further refinement and theme identification. Working with these excerpts, KK assembled analytic summaries of the similarities and differences across the retained and less well-retained groups. These summaries were subsequently compiled into tables (charting). Because we purposefully sampled two theoretically opposing groups with different levels of retention in HIV care, we compared the discussions between these opposing groups. We used the summary tables to identify and refine themes across and within each group (interpretation).

In this analysis we primarily describe the content of *what* was said, however we were also sensitive to *how* it was said, including observing the co-construction of ideas among the participants and facilitators. We represent a few instances of “chaining”—the effect of how ideas were carried forward by group members to illustrate the group dynamic [33]. Note, we did not have permission to re-contact participants for a formal members check to assess the validity of our findings [34] nor did we seek permission to place de-identified transcripts into a public repository.

## Findings

Between February and March of 2014, we conducted six FGDs. Forty-three patients participated in the groups, approximately half in each group: retained (n = 21) and less-well-retained (n = 22). The mean age was 50 years (range 24–71); the majority were men (n = 23) representing a diverse mix of racial and ethnic groups. Table 1 outlines the characteristics of patients.

**Table 1:** Patient Characteristics (N = 43).

Category	Result
*Site*	
Birmingham San Francisco Seattle	*n* = 13 *n* = 14 *n* = 16
*Retention Status*	
Retained Less well-retained	*n* = 21 *n* = 22
Age (median, range)	50 (24–71)
Years since HIV diagnosis (median, range)	19 (4–38)
*Gender*	
Male Female Transgender	23 (53%) 18 (42%) 2 (5%)
*Race*	
African-American Mixed Race/Other White	5 (37%) 22 (51%) 16 (12%)
*Ethnicity*	
Hispanic Non-Hispanic	3 (7%) 40 (93%)

We identified several themes related to the multidimensional concept of engagement in care. First, we noted that participants all appeared to have a common understanding of how a patient engaged in health care should act; often participants implied that an engaged patient was equivalent to a good patient or “someone that follows everything to the tee—you do it the way you’re suppose to.” Whether participants could live up to this idealized patient depiction was related to the presence or absence of narratives reflecting resilience (stories illustrating one’s ability to recover from setbacks). Participant narratives pointed to the variability in how much effort was required to perform the role of the engaged patient.

### Universal internalization of the engaged patient

Participants described what it meant to be well engaged in care with succinct aphorisms such as: “it means being informed,” “smart,” “asking questions,” and “doing what you’re supposed to be doing.” We observed widespread agreement about these otherwise implicit characteristics of a well-engaged patient: an informed, adherent, well organized, self-managing, responsible individual. Members of each FGD, regardless of retention status, geographic location or gender, shared this idealized portrayal of an engaged patient. Attending medical appointments and taking medications as prescribed served as the foundation of behaviors associated with engagement in care.

They expect us to do our part, because we have a part in this thing just like they have a part. Our part is, whatever they provide for us, to utilize it the right way. You know what I’m sayin’–whatever medications they prescribe for us, we take it on time and try to stay healthy, strong, and happy. *Less-well-retained participant*

For me it was very much like being in school, we come to the clinic and we get our regime of medications or what to do and what not to do. And I always want to be above average. So I’ll take five out of seven days of medication. And I had to learn that that’s not how it should be done… We have to adhere to the prescription of the doctor, whether we like it or not. *Less-well-retained participant*

Participants described the well-engaged patient as someone who attends appointments and takes medications in a timely, organized fashion. Correspondingly, when participants were asked to define a patient who was poorly engaged in care, participants pointed to instances of “not following up with doctor, lab work or taking meds regularly.” Less-well-retained patients expressed particularly strong judgments about transgression from the engaged patient role. Behaviors associated with disengagement in care were depicted as personal liabilities such as “they (are) stupid” or “people just don’t care; still smoke dope, drink.” Members of both types of groups could identify occasions when they felt that they performed as disengaged or below average patients; however, the internalization of this sentiment was expressed more frequently among the less-well-retained participants. For example, one person spoke of feeling awkward or “a little shameful” about coming to the clinic for the FGD because he had missed his most recent medical appointment. His story provided insight into the negative psychological consequences of missing appointments—that some patients experience feelings of shame and embarrassment when they miss or avoid appointments and must overcome these adverse perceptions in order to seek future services.

SEAPT03: I know that my doctor is concerned enough to where he calls me to make sure that I make an appointment to see him. But at the same time, too, while he’s done that,I have forgotten appointments, and then (I feel) a little shameful that I had to call to find out that I missed it. Because I’m thinking …when I did miss the appointment? But the shame part of me stopped me from–(coming back). *Less-well-retained participant*

In a separate focus group, we observed participants collectively grapple with defining the parameters of a less engaged patient. We feature this lengthier exchange to illustrate chaining whereby one participant refutes another’s use of the adjective “rebellious” to typify a disengaged patient:

SFPT01: What it (to be not engaged in care) means to me… are you doing the protocol, are you not doing the protocol? To do the protocol means you’re on step…what is assigned to you,–if you tell me I need to take these pills, and I say, no, I don’t, then I’m being rebellious, I’m not listening to what my physician is saying, …that’s not engaged, not doing what he or she is supposed to do.

Facilitator …. Maybe it means not following–you’re not following the rules.

SFPT01: That’s how I look at it.

SFPT02: Well, with my mother, she was going to get health care and she was in a lot of pain from a lot of different disorders, and they wouldn’t give her pain medication because she was an alcoholic, and she refused to stop drinking. And so she continued to drink and eventually it killed her. And so I think that—she did want to get help, but she just couldn’t do what the doctors were asking her to do.

Facilitator: Okay, so back to not following orders and not—yeah. What else does it mean to be not engaged in health care?

SFPT03: For me it was more like—maybe I’m more like that, because I really don’t like to take medicines, I don’t like goin’ to appointments, I’m real hesitant on what the shit you say to me, I don’t trust every doctor, I don’t trust every worker, I don’t trust nobody, pretty much. You gotta earn my trust…. Now, that is just who I am. Does that make me a person that is trying to be rebellious, or am I trying to be who I am and that’s what’s makin’ me comfortable, to be a well-engaged person or–‘cause in my books, I am a well-engaged patient because I doing who I am. … But then, you know, there’s all those missed appointments, there’s a battle just for me to take medicines. Is that well-engaged or is it not?

Facilitator: What do you think?

SFPT03: ‘Cause I feel like I am, I feel like I put some kind of effort into it, so therefore, that makes me a well-engaged patient ‘cause I put the effort into doing something.

The quotes above illustrate patients discerning the extent to which they might consider themselves to be engaged in care. In the first case, this SEAPT03 worried about how he would be received when he entered the care setting after missing a recent appointment. Whether or not the clinic staff would perceive him as an underperforming patient is irrelevant because he assigned that label to himself. He conveyed to us that he fit the description of someone that was not appropriately engaged in care and because of this he expressed feeling ashamed. His examples points to the vulnerabilities patients face that could delay or prevent their access to care. In the second example, SFPT01 marked a definitive boundary: a patient must follow doctor’s orders to be engaged or risk being considered “rebellious” while SFPT02 offered a slightly more nuanced example of why a patient may wish to, but was not be able to comply with doctor’s orders and SFPT03 expressed a sense of indignation about assuming that a rebellious persona automatically implied a lack of engaged. SFPT03 complicated the otherwise rigid description of a less-well-engaged patient as an underperforming patient; she wanted whatever effort she was able to muster to be factored in to the determination of her engagement in care.

### Resilience, vulnerability and the engaged patient

Comparing excerpts from retained and less-well-retained patients showed distinct ways of coping with managing HIV care. The retained participants offered narratives that pointed to resilience, which manifested as tenacity and perseverance. Resilience narratives were expressed as a “I have to make it work” attitude. Retained patients saw ongoing care as both a necessity and one’s personal responsibility to stay healthy:

To be engaged is knowin’ every aspect of what’s going on with your care, to know what’s goin’ on with you, because when you’re first diagnosed, you go through so many feelings… And so I had to come to terms with being HIV positive in order to … forgive myself, in order to take responsibility to be able to help myself, to be able to live every day with this and be able to take care of myself. -*Retained participant*

Less-well-retained patients often expressed greater levels of emotional and physical vulnerability, as well as a level of mistrust in the medical system. This vulnerability and mistrust may have led to decreased desire or ability to ‘hang in there’ with the demands of care and treatment. In some cases, it led to a “I gotta take a break” attitude. Thus, one group articulated resilience, while the other group articulated vulnerabilities in the ability to be adherent to treatment and attend all medical appointments. In this next excerpt, a woman articulated the boundaries she placed on being a “good girl”–she could only sustain this persona for a certain period of time before she needed to withdraw.

I’ve dealt with this for over half my life and there are times when I just get so fed up with all the pills and all the doctor’s appointments. I’m guilty. I just walk away. And I’ll stop dealing—I won’t go to doctor’s appointments and I stop taking my medications just because I gotta take a break. I got to. Because I would like just for a little bit to feel like a normal person. And as long as I have to see the doctor once a month, I have to take those pills three times a day…so being a good girl, I can only do for so long. Granted, I do that. But there are times when I just have to—have to take a break. I just gotta. Then I disengage.–*Less-well-retained participant*

The paradox illustrated in the above case is that in order to stabilize her *mental* health, this participant needed to disengage from HIV care. The burden of managing her HIV appeared to be overwhelming at times, and out of self-preservation, she withdrew from care.

We noted instances of different understandings about the *purpose* of or even the necessity of medical appointments between the groups. Misunderstandings were more common in the less-well-retained group, particularly among participants expressing low health literacy. For example, one participant defined “necessary” medical appointments as “like the labs and the things that need to be done” in contrast to the optional “how you doin’?” appointments. She explained:

There’s not so many appointments people have to go for, other than labs… you don’t have to go for how do you do, how do you feel, with myself anyway. No, ‘How you doin’ (appointments).—*Less–well-retained participant*

In her view, the medical appointments consisting of *conversations* between the patient and provider were unimportant. Another participant questioned why her provider had stopped physically examining her during each appointment, wondering whether he was intentionally withholding hands-on examination or if he was afraid to touch her. These perspectives point to a mismatch in understanding on a basic level about the expectations and motivations for routine clinic visits for people living with HIV. Some patients expected both a discussion about one’s health *and* a physical exam; the experience of being physically examined conveyed that you were not being stigmatized, for one patient or that in the other case, that the provider was performing their job well.

Participants in the less-well-retained groups spoke about clinical encounters with providers with whom they felt incompatible, which made them less likely to want to attend appointments and suggests an area of vulnerability. They described occasions when provider incompatibility led to missing appointments as in the examples below:

I’ve skipped before, because I got this doctor that was kind of a prick. I came in because I was sick with something, and he was really brief with me. And just sent somebody else in to deal with me because I was kind of a mess over it. And so the next time I had an appointment, I didn’t want to come see him. I didn’t come in.–*Less-well- retained participant*

In the above scenario, the participant intimates that her provider’s response to her being a “mess” was to ask someone else to step in and provide care. She explained to the group that she perceived that her provider was judgmental about her drug use and held her personally responsible for her “messy” situation. Another member of the FGD chimed in with a story about incompatibility with someone that was not his usual provider:

I came here and saw another doctor …and she’s young, and "Are you homeless, by the way?" I said, "No, are you?" And I just said, "You know what? Screw you." I said, "I’ll see somebody else. I don’t need to be seen today." And I left…. I guess the whole thing was she was talking down to me. And once you talked down to me, I’m out of there I don’t care who you are.—*Less-well-retained participant*

Participants spoke of their preference to be in a long-term relationship with a provider who knew them well. Participants in the less-well-retained groups often shared stories about perceiving instances of judgment and disrespect while seeking HIV care. Urgent care appointments with or without one’s assigned provider delivering care may be situations where vulnerability about one’s status as a ‘good’ or ‘bad’ patient may be particularly heightened.

### Experiencing engagement in care as work

Patient perceptions of the burden of health care and time spent in care are important in understanding how patients experience health, illness and care. While members of both groups described instances of engagement in care as burdensome, the less-well-retained patients experienced HIV care as burdensome to a higher degree than retained participants. However, it was a retained participant who most clearly articulated the psychological effects of the energy and hours required of patients living with HIV to engage in care over the lifespan. This participant drew our attention to something that is frequently underemphasized in the literature on retention in HIV care: the effort required on the part of the patient to attend appointments and to follow treatment recommendations.

Until my first job I had not had any experience of doctors, because I never needed the services of a physician… then a blood test popped up positive. So now I deal with health care…. it’s an extremely distressing experience…. coming to the doctor and making three different appointments to deal with one issue, and understanding the whole process, I came into it cold, basically, so I didn’t know what I was facing… it becomes overwhelming, because you are constantly seeing a doctor,… you look at the rest of your life, and that’s what you see, is appointment after appointment after appointment.—*Retained participant*

In contrast to the example above, another participant described his ready submission to “health work” by evoking many of the ‘good’ patient characteristics described earlier. He perceived the work as his personal responsibility i.e., “it’s not your doctor’s life, it’s your life,” and appeared to be less concerned about the effort required on his part, expressing a greater ability to persevere than the previous participant when faced with challenges associated with engaging in HIV care over time. He explained:

I can describe it (engagement in care) in three words—well-informed consumer… what that means is it’s doing your homework, it’s learning all you can about the virus, about the drugs, about the research, so you can make an informed decision…. it’s knowing that doctors … have the best of intentions, but you have to be a partner with them, you have to do the work, because it’s not your doctor’s life, it’s your lif… Engagement means getting in there and doing the work, because when I finally decided I was ready to live, I came up with the two types of people in the world who have HIV. That are those that are in denial and don’t want to deal with it, and they die. And then there’s everybody else, who says, damn it, I want to live, and they do the work and they fight for life, and they fight for the things they want …So that to me is engagement. But that takes a lot of work from us. *Retained participant*

This patient rationalized the work involved in HIV care—this work, in his view, meant he stayed alive. He simplistically argued that people living with HIV had two options, to engage in care and live or to not engage and die. This view was in stark contrast to the female patient who spoke of needing a break from HIV care and saw appointments and medications as an overwhelming burden, a burden which to her signified an abnormal state. She pulled away from care to experience a sense of normalcy, which for her meant daily life free from medications or medical appointments. One member of the Seattle retained focus group pushed back on the premise that you have to be fighting for oneself. In his experience, he was too sick to self-advocate and instead needed someone else to advocate on his behalf noting, “if you don’t have it and have to reach out and find (an advocate). That’s hard.” Expresssions of vulnerability such as this one did come up for people in retained groups, just far less often than in less-well-retained groups.

The idea of care fatigue or being “fed up” was rarely present in narratives of those who described engagement in care as a personal responsibility they willingly took on. This depiction of care engagement as burdensome by less-well-retained patients makes sense in light of the presence of vulnerability among patients with fewer material and emotional resources to draw on than retained patients. Under these conditions, patients may be prone to see themselves as ‘bad’ patients and unable to achieve ‘good’ patient status.

## Discussion

In this analysis, we examined patient perspectives on engagement in care by comparing the experiences and definitions of HIV care engagement between a group of retained and less-well-retained patients, as defined by medical appointment attendance and viral suppression. We noted a recurring pattern in the responses to the question about the meaning of engagement in care, Participants routinely constituted the *engaged* patient as a *good* patient meaning that inevitably when we asked about the meaning of an engaged patient, participants imposed a moral framework on enagement and its opposite, the bad patient. Social scripting theory may be a useful guide to further interpret the FGD narratives [35]. Social scripting is a metaphor used to convey the production of behavior and practices within social life [35] and can be used to study tacit expectations of how to comport oneself while performing any number of roles, including that of the patient.

Underlying the concept of patient engagement is an aspirational image of a ‘good’ patient. The literature on the social construction of this phenomenon, while limited, describes the attributes of a ‘good’ patient script as a person who is willing to adhere to a care plan, is demonstrably appreciative of services, and is coping well with a diagnosis [36]. The degree to which patients adhere to the ‘good’ patient script is variable and while some may find the structure of the script to be easy to adhere to, others may not. Here, we articulate the mechanisms by which the ‘good’ patient phenomenon shapes patient engagement. These findings can be leveraged to understand engagement in HIV care from a nuanced social perspective.

We found that well-retained and less well-retained groups assigned a moral dimension to engagement in care, in that well-engaged patients were considered to be ‘good’ patients. A key difference between the groups included stronger narratives of resilience among the retained participants, and greater levels of vulnerability to disruptions in care among the less-well-retained patients. While aspiring to become a ‘good’ patient provided a meaningful goal for some patients, this same concept deepened vulnerabilities among patients who struggled to achieve this status. It appears that more vulnerable patients may feel less secure in health care interactions and that these feelings of insecurity may be amplified if patients have an unreasonable impression of what constitutes a ‘good’ patient; thereby leading to disengagement in care.

Relatedly, we noted differences in how the two groups perceived the activity of engaging in health care. Less-well-retained patients viewed health care activities—appointments, taking medications, following up on referrals—as burdensome and at times, overwhelming. This perspective of healthcare as burdensome rather than as a manageable task may influence one’s level of engagement in care. Less-well-retained patients expressed greater levels of emotional and physical vulnerability and occasionally a mistrust in the provider, which led to less ability or desire to ‘hang in there’ with the demands of care engagement. Many expressed a “I gotta take a break” attitude and assumed that the ‘good’ patient status was out of reach for them, especially participants who expressed limited health literacy, feelings of incompatibility with their provider, frustration about medications and appointments, or shame about missing appointments—attitudes that are antithetical to the ‘good’ patient ideal. In contrast, the qualities of a ‘good’ patient appeared to require far less effort to enact among retained participants, many of whom still perceived these qualities as their “work.”

Existing literature and commentary on the concept of the ‘good’ patient offers insight into what patients seek to lose or gain when they meet the standards of the ‘good’ patient. One study of patient attitudes on shared-decision making among upper middle-class patients reported that patients strategically avoided participating in shared decision-making for fear of threatening their standing as a ‘good’ patient in the eyes of their provider [37]. Specifically, asking questions could be too time-consuming for the provider and may be interpreted as a challenge to the provider’s authority. Another study examining how notions of the ‘good’ patient affected patient-nurse relationships and to antiretroviral therapy (ART) adherence in Zimbabwe found that the main reasons to be a ‘good’ patient were to ensure receipt of quality care and on-going access to ART [38]. Patients who did not fit the ‘good’ patient persona were reprimanded by nurses and found clinic visits to be “highly stressful and discouraging” and study authors hypothesized that such experiences likely resulted in avoidance of care [38]. Our analysis supports the hypothesis that people who see themselves as bad patients are susceptible to disengagement in care.

Higgins and colleagues argue that defining the broader concept of patient engagement may lead to higher quality care for patients [2]. With greater understanding of the four central attributes of patient engagement, clinic administrators and clinicians might generate interventions to support the concept. While our research was not directly informed by these defining attributes, the recommendations stemming from our work can be usefully framed within the domains of *personalization—*the tailoring of interventions based on the context and life circumstances of a particular patient, *access*—a patient’s ability to seek and receive resources, medical and otherwise, *commitment*–the cognitive and emotional factors that motivate patients to access available resources and the *therapeutic alliance* the possibility for an equitable rather than a hierarchical patient-provider partnership [2].

### Implications

Ideally, our findings provide insight into how the ‘good’ patient persona influences care engagement. Our analysis indicates that negative self-judgment takes place among all patients, but at particularly high levels among the less-well-retained patients and should not be underestimated. Even a small antagonism, pressure or judgment on the part of the clinic or the provider perceived by the patient, intentional or unintentional, can make a significant impression on patients. Given these insights, we offer recommendations that may help foster positive relationships between patients and clinic staff.

#### Help patients see themselves differently

We recommend that healthcare providers directly address the stereotype of the bad patient by debunking unreasonable standards and myths about what it means to be a ‘good’ patient. Communicating with less-well retained patients in this way is an example of a *personalized approach to care* or tailoring to the unique circumstances of a patient that constitutes one of the pillars of patient engagement. For example, providers might normalize the occasional missed appointment, missed doses of medication or mention the possibility of becoming “fed up” with pill-taking. This would be particularly valuable for, but not limited to, patients newly diagnosed with HIV. Finding ways to leave the door open for patients who have feelings of inadequacy, or those that feel they have disappointed their provider, could help facilitate engagement. Patients often overlook the implicit strategies they undertake to manage their health; identifying and providing positive feedback on the ways in which patients are self-managing is one way to help patients to see themselves differently.

#### Address the dichotomy of ‘good/bad’ patient

If patients suffer from feelings of inadequacy around their ability to perform the ‘good’ patient persona, clinic staff and providers may be inadvertently exacerbating this problem. In fact, one study found that providers characterized only a modest proportion of patients (approximately one-third) as fitting the definition of a person interested in and responsibly participating in their care [39]. These findings suggest that providers may categorize the majority of their patients as performing at a suboptimal level. We recommend that clinic staff and providers periodically reflect on expectations they hold for patients, both in general and specific cases, to consider whether these expectations are reasonable. Punitive administrative policies, such as refusing care to those with multiple missed appointments or charging fees to those who no-show, may reinforce patients’ feelings of inadequacy and be counterproductive. For patients newly diagnosed with HIV, it may be particularly important to address feelings of failure and stigma associated with seroconversion. Having clinic staff/and or a provider directly address some of these fundamental feelings may be an impactful gesture, especially given the power (and perhaps social status) differential between a respected provider and a patient grappling with a diagnosis of a socially stigmatizing illness. For providers who may be uncomfortable directly bringing up stigma, which can be a difficult topic to initiate, according to Fredericksen and colleagues, patients suggested that providers ask patients about….. “social and familial adjustments” in order to indirectly approach the topic of stigma [40]. Patients implied that this line of questioning would be demonstrative of support and understanding by their providers.

#### Develop supportive systems that facilitate patients’ ability to succeed

For example, case managers and/or social workers might create agreements with patients on how to handle missed appointments to avoid patients’ self-blame or negative thinking. As in our previous work, we recommend the development of patient educational materials explaining the roles, responsibilities and expectations of the clinic staff, providers, and patients in the context of HIV care [27]. This may include the following as applicable: 1) clinic staff are expected to e.g., schedule appointments, call to remind patients of appointments, facilitate access to benefits programs, social work, mental health services, case management, care coordination etc.; 2) providers/provider teams are expected to e.g., perform an annual physical exam, explain that a physical exam is indicated annually and as symptoms indicate, but not necessarily at every visit, and that visits which consist of discussions of adherence, barriers to adherence, etc. are also important, ask and answer questions, and be open to but not insist on shared-decision making and 3) patients are expected to e.g., present for ongoing laboratory monitoring, ask and answer questions, share in decision-making as warranted, etc. Patients could also be asked to describe their expectations and preferences with regard to communication styles e.g., frank or “sugar-coated” communication, with an understanding that expectations and preferences can change over time. A checklist of questions or statements could guide the conversation. For example, patients could be asked whether they prefer to be involved in decisions at every level versus a preference to have the provider take the lead on decision-making based on her/his expertise. Furthermore, to manage encounters with sensitive patients who may struggle with a ‘bad’ patient persona, a provider or care team member might explicitly remind a patient that they have their best interests in mind and do not intend to embarrass them or induce feelings of shame.

## Conclusion

Consistent engagement in HIV care requires endurance, organization and resources, and medical appointments may have different meanings and be differently valued by patients with varying levels of these capacities. These findings have implications for developing clear messages to patients with intermittent appointment attendance. A ‘good’ or ‘bad’ patient persona can be shifted by introducing patients to a different set of ideals to work towards, particularly if these ideals and goals that tailored to and are within reach of a specific patient. Finding ways to communicate reasonable expectations regarding the management of their HIV, specifically appointment attendance, as well as medication adherence, could alleviate some of the burden experienced by less retained patients.

The importance of understanding patients’ explanations of engagement in care fostered recognition about patients’ predilection to harshly judge their personal shortcomings. Our data suggest we promote expectations for patient engagement that are reasonable, rather than perfect. Recognizing that self-blame may be causing patients to remain outside of the care system could potentially be mitigated with messages about how patients might achieve a state of wellbeing that is specific to their context. Messages that communicate a personalized version of wellbeing may be helpful for people living with HIV who struggle with process of being fully engaged in HIV care.
